# Id-1: Regulator of EGFR and VEGF and potential target for colorectal cancer therapy

**DOI:** 10.1186/1756-9966-27-69

**Published:** 2008-11-12

**Authors:** Ibrahim Meteoglu, Nezih Meydan, Muhan Erkus

**Affiliations:** 1 Adnan Menderes University, Medical Faculty, Department of Pathology, 09100-Aydin/Turkey; 2 Adnan Menderes University, Medical Faculty, Department of Medical Oncology, 09100-Aydin/Turkey

## Abstract

**Background:**

The helix-loop-helix transcription factor Id-1 (an inhibitor of differentiation and DNA binding) plays a role in development and progression of many tumours. Id-1 is known to exert its effects on the epidermal growth factor receptor (EGFR) and the vascular endothelial growth factor (VEGF). The aim of this study was to reveal whether there was a relationship between Id-1 and EGFR and VEGF in colorectal carcinoma.

**Methods:**

Tumour and non-tumour tissue specimens from 46 cases of colorectal carcinoma were exposed to immunohistochemical staining for Id-1, EGFR and VEGF. The relationship between the degree of staining and tumour grade, tumour stage and all tumour markers was investigated.

**Results:**

Tumour cells showed positive staining for Id-1 in 43 cases (93.5%), for EGFR in 41 cases (89%) and for VEGF in 42 cases (91%). There was a significant relation between the tumour grade and the degree of staining for Id-1, EGFR and VEGF. The relation between the tumour stage and the degree of staining for Id-1, EGFR and VEGF was also significant. There was a significant relation between Id-1 expression and EGFR and VEGF expressions. Non-tumoural tissue specimens were not stained with Id-1 and EGFR antibodies in any of the cases, but stained with VEGF antibody in 3 cases.

**Conclusion:**

This study revealed that Id-1, EGFR and VEGF took part in development and progression of colorectal carcinomas and that Id-1 was associated with regulations of EGFR and VEGF. The results of this study support the idea that not only EGFR and VEGF but also Id-1 could be new targets in cancer treatment.

## Background

Colorectal carcinomas are frequently encountered tumours in the world, especially in developed countries. These tumours account for about 10% of deaths from cancer [1]. They were the second and the third most frequent causes of deaths from cancer in males and females respectively. The conventional treatment for colorectal tumours is surgery. Adjuvant treatment is employed for advanced stage tumours.

It is known that tumour grade, penetration depth, angiolymphatic invasion and lymph node involvement are important conventional parameters used to determine the prognosis in colorectal tumours [2]. However, histopathological parameters do not exactly show the prognosis in some cases and therefore, additional prognostic factors are needed. In recent years, there have been many studies on new markers to predict the prognosis and to determine tumours resistant to treatment.

It has been reported that epidermal growth factor receptor (EGFR) expression is associated with tumour proliferation, angiogenesis and invasiveness in many tumours [3]. One of those tumours is colorectal carcinoma [4]. In fact, EGFR has been shown to play a role in tumour development and progression [4,5]. In addition, several studies showed a relationship between high EGFR levels and high grade tumours and poor prognosis [5,6]. The studies on EGFR have suggested that EFGR can be a new target molecule in anticancer treatments.

Angiogenesis has a critical role in tumour progression. There are many factors known to be associated with angiogenesis. Vascular endothelial growth factor (VEGF), the most important and most extensively studied factor, is known to be associated with many tumours [7]. VEGF has been shown to be associated with the extent and the stage of colorectal carcinomas [8].

Several studies, however, have revealed that VEGF is not an independent prognostic factor in colorectal carcinomas [8,9]. The Id (an inhibitor of differentiation/DNA binding) protein family, a group of basic helix-loop-helix transcription factors, has been shown to be involved in carcinogenesis and a prognostic marker in several types of human cancers [10-16]. Id-1 does not have a basic domain for DNA binding and it forms heterodimers and acts as a dominant inhibitor of simple helix-loop-helix transcription factors [17,18]. Id-1 has been shown to play a critical role in cell proliferation, differentiation and senescence. It has been claimed to play a key role in cancer progression, but its molecular mechanism has not been understood yet. Increased Id-1 expression has been found to be associated with advanced stage tumours and poor prognosis. In addition, ovarian cancer patients with increased Id-1 levels have shorter survival than those with low Id-1 expression [19]. High Id-1 levels have been shown to be associated with more than 20 types of cancer including breast, prostate, cervical, liver and endometrial cancer [20-26]. It has been shown that Id-1 is constitutively expressed in aggressive cells in breast cancers [11]. High Id-1 levels have also been found to accompany high grade and invasive endometrial cancers [22]. Increased Id-1 levels have been considered as the indicator of more aggressive clinical behaviour in breast and cervical cancers [11,14-16]. It has also been demonstrated that increased Id-1 levels are accompanied by high Gleason scores in prostate cancers [27]. These results indicate that Id-1 may be involved in not only carcinogenesis but also several types of cancer.

Recently, it has been proposed that Id-1 is a regulator in the EGFR and VEGF pathway [28-30]. Id-1 mediated cancer cell growth has been shown to be associated with EGFR activation in ovarian and prostate cells [19,24]. Both Id-1 and EGFR levels have been found to be high in bladder cancers [29]. These results indicate that Id-1 plays a role in cancer cell growth through positive regulation of the EGFR signalling pathway. It has been reported that overexpression of Id-1 in prostate cancer cells play a role in angiogenesis whereby VEGF activation [30]. Id-1 suppression, however, has been found to down-regulate VEGF in hepatocellular carcinoma [20].

In the light of all above mentioned studies, one can conclude that Id-1 has an important role in progression of many tumours. However, there have been few studies on the role of Id-1 in colorectal carcinoma [10,31]. Those few studies have revealed that Id-1 is involved in tumour progression and associated with poor prognostic factors, but the relationship between Id-1 and EGFR and VEGF has not been studied. In this study, using immunohistochemical methods, we investigated the relationship between Id-1 and such well-known prognostic factors as tumour grade and tumour stage and such molecules as EGFR and VEGF.

## Methods

### Patients

This study included 51 patients who underwent surgery for colorectal cancer in Adnan Menderes University Hospital between 2002 and 2006. Five patients were excluded from the study since data about those patients were incomplete. Data about sex, age, tumour size, TNM and Dukes' stage were obtained from all patients. None of the cases received treatment for cancer before surgery. All specimens were re-evaluated before the current study by two pathologists. Paraffin-embedded specimens that contained normal colonic tissue together with tumour tissue were selected for immunohistochemical analysis.

### Immunohistochemistry

First, sections 4 μm in thickness from selected paraffin embedded blocks were placed on positively charged slides. Second, all of the specimens were deparafinized with xylene and rehydrated with ethanol and then all were washed in 0.1% Tris-buffered saline (TBS) for five min three times. Third, they were kept in 0.3% hydrogen peroxidase for 20 minutes for the blockage of endogenous peroxidase activity and the sections were rinsed in TBS for five min three times. Fourth, for antigen retrieval, the slides were placed in sodium citrate buffer and left in a microwave oven at 700-W for 10 minutes. After cooling to room temperature and washing with TBS for five min three times, non-specific binding was blocked with 10% normal rabbit serum for 1 hour. Fifth, they were incubated with primary antibodies Id-1 (1:400 dilution, Santa Cruz Biotechnology, Santa Cruz, CA, USA), VEGF (ready-to-use solution, RB-9031; NeoMarkers, Fremont, CA, USA) and EGFR (ready-to-use solution, MS-378; NeoMarkers, Fremont, CA, USA) for 1 hour. Sixth, after these the slides were rinsed with TBS for five min three times, they were exposed to biotin free horseradish peroxidase (HRP) enzyme-labelled polymer (EnVision plus the detection system, ChemMateTM EnVision +/HRP Rb&Mo, DAKO, Hamburg, Germany). Thirty minutes later, the slides were rinsed again in TBS for five min three times. Then 3,3'-diaminobenzidine (DAKO) chromogen substrate was added. Last, the slides were rinsed under tap water and countered with Mayer's haematoxylin, dehydrated and mounted. For the negative control, the slides were incubated with TBS without primary antibody. The slights known to be positively immunostained were used as positive controls.

### Evaluation of the immunostaining

The slides were examined at low magnification (×100) and areas containing the highest degree of staining were considered as "hot spot" areas and evaluated at high magnification (×200). The staining cell ratio was determined by counting at least 200 cells. When 10% or more than 10% of the cells were stained with all the markers, staining was considered positive. The staining degree in different magnifications were evaluated as in the following: "weak" staining visible at × 200, "moderate" staining visible at × 100 and "strong" staining visible at × 40.

### Statistical analysis

All statistical analyses were performed with SPSS (Windows version 13.0, SPSS Inc. Chicago, IL, USA). χ^2 ^and Mann-Whitney *U*-test were used to compare the parameters tested and Spearman's correlation analysis was used to determine the relation between immunohistochemical markers. P < 0.05 was considered significant.

## Results

The patients' clinicopathologic characteristics are summarized in Table 1.

**Table 1:** Patients characteristics (n = 46)

	n (%)
Age (year)	
Range	33–94
Median	62
	
Sex	
Male	29 (63)
Female	17 (37)
	
Dukes' stage	
A	7 (15.2)
B	12 (26.1)
C	11 (23.9)
D	16 (34.8)
	
Grade	
G1	10 (21.7)
G2	21 (45.7)
G3	15 (32.6)

### Staining for Id-1, EGFR and VEGF in Tumoural and Non-tumoural Tissues

**Figure 1:**
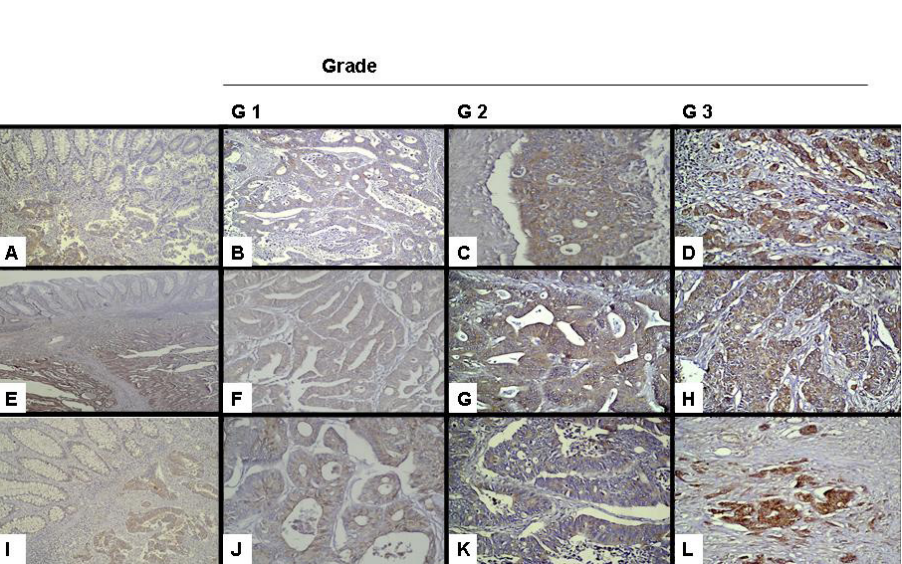
**Differential staining intensity of Id-1, EGFR and VEGF between non-tumoural colorectal tissues and colorectal carcinoma specimens (A-E-I).** 2-Images of Id-1 (B-C-D), EGFR (F-G-H) and VEGF (J-K-L) expressions in different grades of colorectal carcinoma. Note that increased Id-1, EGFR and VEGF expressions are associated with increased tumour grade.

Out of 46 cases, 43 (93.5%) had positive cytoplasmic staining with Id-1 antibody. Most of them had moderate to strong staining. Forty-one cases (89%) had moderate to strong staining with EGFR antibody in the cytoplasm and cell membranes. Forty-two cases (91%) had moderate to strong staining with VEGF antibody. There was no staining with Id-1 and EGFR antibodies in non-tumoural tissues, but there was stromal staining with VEGF antibody in three cases (Figure 1A–E–I).

### Relationships between Tumour Grades and Id-1, EGFR and VEGF

In order to investigate the relation between colorectal tumour grades and Id-1, EGFR and VEGF, the tumours were divided into three groups: grade I (G I) (n = 10), grade II (G II) (n = 21) and grade III (G III) (n = 15) tumours. Most of the cancer specimens had positive staining for Id-1, EGFR and VEGF. G III tumours had the highest degree of staining for Id-1, while the degree of staining was lower in G I tumours. As a result, there was a significant difference in Id-1 expression between G I and II tumours (p = 0.005) and between G II and III tumours (p = 0.0012) (Figure 1B–C–D). The degrees of staining with EGFR and VEGF antibodies were very similar to the degree of staining with Id-1 antibody and as tumour grade increased so did the degree of staining. There was also a significant difference in EGFR (Figure 1F–G–H) and VEGF (Figure 1J–K–L) expressions between G I and II tumours (p = 0.02 and p = 0.05, respectively) and between G II and III tumours (p = 0.01 and p = 0.04, respectively) (Table 2).

**Table 2:** Association between Id-1, EGFR, VEGF, tumour grade and Dukes' stage.

	**Grade**			**Dukes' Stage**	
	
	G1(n = 10)	G2 (n = 21)	G3 (n = 15)	A+B (n = 19)	C+D (n = 27)
**Id-1**					
Absent	2 (20%)	1 (4.8%)	0 (0%)	3 (15.8%)	0 (0%)
Weak	3 (30%)	9 (42.8%)	1 (6.7%)	2 (10.6%)	3 (11.1%)
Moderate	5 (50%)	8 (38.1%)	5 (33.3%)	6 (31.5%)	10 (37%)
Strong	0 (0%)	3 (14.3%)	9 (60%)	8 (42.1%)	14 (51.9%)
					
*p*-value	*p *= 0.005 *(G1 versus G2)*		*p *= 0.0012 *(G2 versus G3)*	*p *= 0.018	

**EGFR**					
Absent	3 (30%)	2 (9.5%)	0 (0%)	5 (26.4%)	0 (0%)
Weak	3 (30%)	8 (38.1%)	2 (13.3%)	3 (15.8%)	5 (18.6%)
Moderate	3 (30%)	7 (33.3%)	3 (20%)	4 (21%)	9 (33.3%)
Strong	1 (10%)	4 (19.1%)	10 (66.7%)	7 (36.8%)	3 (48.1%)
					
*p*-value	*p *= 0.002 *(G1 versus G2)*		*p *= 0.001 *(G2 versus G3)*	*p *= 0.03	

**VEGF**					
Absent	3 (30%)	1 (4.7%)	0 (0%)	4 (21%)	0 (0%)
Weak	2 (20%)	7 (33.3%)	1 (6.7%)	3 (15.8%)	5 (18.6%)
Moderate	2 (20%)	8 (38.1%)	3 (20%)	5 (26.4%)	10 (37%)
Strong	3 (30%)	5 (23.9%)	11 (73.3%)	7 (36.8%)	12 (44.4%)
					
*p*-value	*p *= 0.05 (*G1 versus G2)*		*p *= 0.04 *(G2 versus G3)*	*p *= 0.04	

### Relationships between Tumour Stages and Id-1, EGFR and VEGF

In order to investigate the relation between tumour stages and staining with Id-1, EGFR and VEGF antibodies, the tumours were divided into two groups based on Dukes' classifications: Group 1 (Dukes' stage A+B, n = 19) and Group 2 (Dukes stage C+D, n = 27). The degree of staining for Id-1 was significantly higher in Group 2 than in Group 1 (p = 0.018). Similarly, EGFR and VEGF expressions were significantly higher in high stage tumours than low stage tumours (p = 0.03 and p = 0.04, respectively) (Table 2).

### Relationships between Id-1, EGFR and VEGF Expressions

Id-1 expression was significantly correlated with EGFR expression (r = 0.540, p = 0.002) and VEGF expression (r = 0.737, p = 0.0001). However, there was no significant difference between EGFR and VEGF expressions.

## Discussion

Colorectal cancer was the second most frequent cause of death in men and the third most frequent cause of death in women [1]. Colorectal carcinomas are more frequent in developed countries and there have been a lot of studies on the disease. The studies have focused on such issues that help to predict the prognosis and provide guidance in treatment. There is strong evidence that EGFR and VEGF play a role in carcinogenesis and progression in many human cancers. Recent studies seeking new treatment alternatives for advanced stage tumours have shown that EGFR and VEGF are new targets [32,33]. Id-1, a negative regulator of basic helix-loop-helix transcription factors, plays an important role in the regulation of cell proliferation and differentiation. Id-1 is itself not a transcription factor, but involved in regulation of many regulatory proteins [17]. Although Id-1 is thought to play a role in tumorigenesis and can be a new therapeutic target, it is not clear how it affects molecular mechanisms in various tumours. There have been a few studies on the relation between Id-1 and colorectal carcinoma [10,31]. However, to our knowledge, this study was the first to show a relation between Id-1 and EGFR and VEGF in colorectal cancers.

It is known that EGFR is involved in carcinogenesis in many tumours. It plays a role in many events such as tumour proliferation, angiogenesis and invasiveness [3-6]. While some studies have revealed that EGFR is associated with poor prognosis in colon cancers, other studies have shown that there is no relationship between EGFR and survival [3]. We found that EGFR was significantly associated with high grade and high stage tumours. This finding indicates a role of EGFR in tumour progression.

Like EGFR, Id-1 has been shown to have an up-regulation in many tumours [19,24]. Increased Id-1 levels are associated with poor prognosis in advanced stage tumours [22,23,27]. It has been reported that Id-1 did not have an activity in normal tissue in some tumours, but was only expressed in cancer cells [28]. Therefore, it has been suggested that Id-1 can be considered as an oncogene. There have been many studies showing a relationship between Id-1 and EGFR in many types of cancer. Studies on prostate cancer, ovarian cancer and renal cell carcinoma showed high Id-1 levels to be correlated with high EGFR levels [19,24,28]. Zhang et al. reported that Id-1 inactivation suppressed EGFR expression in ovarian cancer cells [19]. These findings have suggested that Id-1 may play a role in the regulation of EGFR. Consistent with the results of previous studies, this study revealed that increased Id-1 expression was correlated with high grade and advanced stage tumours and that Id-1 expression was also significantly associated with EGFR expression. The significant association between high Id-1 levels and high EGFR levels suggested a coactivation of EGFR and Id-1. It is thought that EGFR is one of the newest therapeutic targets for many tumours. Galizia et al. reported that antiEGFR chemotherapeutics could be used as an adjuvant therapy in EGFR positive colon cancer patients [35]. The finding that Id-1 plays a role in the EGFR pathway, an alternative likely to be used in advanced colorectal cancers, indicates a new therapeutic target.

Angiogenesis is of great importance in cancer progression. Many angiogenic molecules have been described and the most important and the most extensively studied of all is VEGF. VEGF is a key molecule in normal and abnormal angiogenesis. It is known that many factors play a role in regulation of VEGF. VEGF in tumoral and non-tumoral tissues is mainly regulated by hypoxia. Apart from hypoxia, many cytokines, hormones and growth factor regulates VEGF expression in various tissues. Many factors such as EGF, TGF-Beta, IL-1alpha, Insulin-like growth factors (IGFs), TSH and ACTH are autocrine and paracrine regulators of VEGF expression. VEGF has been shown to be involved in many types of cancer [7]. Although it has been reported to be associated with poor prognosis in many types of cancer, there is not an agreement about its association with poor prognosis in colorectal cancers [8,9]. There is a general agreement that VEGF expression is an indicator of poor prognosis. Although there have been many studies about VEGF, VEGF regulated mechanisms have not been understood well. In this study, VEGF was significantly associated with high grade and advanced stage colorectal cancer. It turned out to contribute to tumour progression. There have been few studies showing a relation between Id-1 and VEGF. Those studies revealed a correlation between increased Id-1 levels and increased VEGF levels in hepatocellular carcinoma and prostate carcinoma [20,30]. However, the role of Id-1 in angiogenesis has not been clearly understood yet. There have been studies showing that Id-1 leads to transcriptional activation of VEGF whereby stabilization of hypoxia inducible factor-1alpha (HIF-1alpha) protein [20,36]. In this study, high Id-1 expression was significantly associated with high VEGF expression in colorectal carcinomas, which is consistent with the results of the previous studies on different types of tumours. Like EGFR, VEGF is a new therapeutic target [7]. Although it is still debatable whether angiogenesis affects advanced colorectal carcinomas, we found that increased angiogenesis, shown by VEGF, contributed to tumour progression and that Id-1 was associated with VEGF in colorectal carcinomas. Our study revealed that Id-1, not alone but in combination with other factors, play a role in VEGF activation. Further studies are needed to elucidate complex relationships of factors in tumour angiogenesis.

There have been few studies showing the role of Id-1 in colorectal carcinoma. Wilson et al. reported that Id proteins increased in cancerous tissue in cases of colorectal cancer and that increased Id proteins were associated with mitotic index and p53 expression [31]. Zhao et al. found marked Id-1 expression in cancer cells in cases of colorectal carcinoma compared to normal mucosa and adenomas [10]. They reported that Id-1 expression was higher when Dukes' stage increased and when there was a lymph node metastasis. Likewise, in this study, Id-1 expression was associated with poor prognostic factors. In addition, no Id-1 expression was detected in the normal colon mucosa neighbouring the tumour. These findings support the idea that Id-1 played a role in the induction and progression of colorectal carcinoma.

## Conclusion

Increased Id-1 expression was associated with high grade and advanced stage colorectal carcinomas, which suggested that Id-1 could be used as a prognostic indicator. As far as we know, this is the first study to show that Id-1 expression was significantly associated with EGFR and VEGF in colorectal carcinomas. The finding that Id-1 had effects on the molecules EGFR and VEGF in colorectal carcinomas is important in that these two molecules have been considered as new therapeutic targets for many advanced tumours recently. Detection of new proteins such as Id-1 exerting their effects on both EGFR and VEGF will reveal alternative therapeutic targets. When these molecules are used in treatment, the pathways such as EGFR and VEGF contributing to tumour progression through different mechanisms will be kept under control by a single therapeutic agent. In addition, absence of Id-1 in non-tumoural tissues suggests that systemic chemotherapeutic agents may not affect normal cells and that toxic damage due to chemotherapy may not occur. However, further studies are needed to confirm the results of this study. The immunohistochemical technique we used in this study is simple and inexpensive and allows determining important molecules likely to be prognostic indicators and new therapeutic targets. If used frequently, evaluation criteria will be developed and more research on these indicators likely to be used in routine pathology reports will be made.

## Competing interests

The authors declare that they have no competing interests.

## Authors' contributions

IM conceived of the study and wrote the manuscript. ME and NM participated in the design of the study and helped write the paper. All authors read and approved the final manuscript.
